# Synergistic effects of obesity and type 2 diabetes on adiposity-related cancer risk across age strata

**DOI:** 10.1186/s12916-026-04842-8

**Published:** 2026-04-13

**Authors:** Alex E. Henney, Megan Heague, Uazman Alam, Daniel J. Cuthbertson

**Affiliations:** 1 Department of Cardiovascular and Metabolic Medicine, University of Liverpool, Liverpool, UK; 2 Metabolism and Nutrition Research Group, Liverpool University Hospitals NHS Foundation Trust, Liverpool, Merseyside UK; 3 Liverpool Centre for Cardiovascular Sciences, University of Liverpool and Liverpool University Hospitals NHS Foundation Trust, Liverpool, Merseyside UK; 4 Centre for Biomechanics and Rehabilitation Technologies, Staffordshire University, Stoke-On-Trent, UK

**Keywords:** Obesity, Diabetes, Cancer, Adiposity

## Abstract

**Background:**

Obesity and type 2 diabetes (T2D) each independently increase cancer risk, but their combined impact is less understood. With rising early-onset obesity, T2D, and cancer in younger populations, we assessed how diagnoses of both obesity and T2D affect adiposity-related cancer incidence compared to each condition alone, focussing on age-specific trends.

**Methods:**

We performed a retrospective (real-world) cohort analysis in a large global federated database (TriNetX, Cambridge MA, USA). Three cohorts were generated and compared with a reference arm, patients without obesity or T2D: cohort 1—patients with obesity, without T2D; cohort 2—patients with T2D, without obesity, and cohort 3—patients with both obesity and T2D. Cohorts underwent propensity score matching (PSM) 1:1 of confounders. We examined 5-year rates of incident cancer (including thirteen (traditional) adiposity-related cancers and an expanded list of 24 adiposity-related cancers), performing stratified analyses by age (younger, middle-aged, and older adults (< 40, 40–60, > 60 years), respectively), ethnicity (white, and non-white), and sex (male and female).

**Results:**

After PSM, we compared 2,655,891 people with only obesity, 290,965 with only T2D and 705,499 people with both obesity and T2D, against the reference (1:1). The highest risk was observed in patients with both obesity and T2D (traditional cancers (HR 1.48 [95% CI 1.15, 1.51]) and all adiposity-related cancers (1.30 [1.27, 1.32])). T2D increased the risk of both traditional (1.35 [1.31, 1.39]) and all adiposity-related cancers (1.27 [1.23, 1.31]) to a greater extent than obesity only (increased risk of traditional adiposity-related cancers (1.07 [1.05, 1.09]), but not all adiposity-related cancers (0.99 [0.98, 1.01]). Concerningly, the highest risk of all traditional and adiposity-related cancers was seen in younger (< 40 years) adults with obesity and T2D (1.40 [1.10, 1.79] and 1.58 [1.21, 2.05], respectively).

**Conclusions:**

Risk of incident adiposity-related cancer is driven most strongly by the combination of obesity and T2D, versus either alone, across all age groups, including those with early-onset disease. The impact of early-onset obesity and T2D provides a critical public health problem that demands targeted screening and management.

**Supplementary Information:**

The online version contains supplementary material available at 10.1186/s12916-026-04842-8.

## Background

The obesity pandemic has driven the incidence of numerous obesity-related complications including type 2 diabetes (T2D), metabolic dysfunction-associated steatotic liver disease (MASLD), cardiovascular disease (CVD) and cancer with reductions in quality of life, and significantly increased morbidity and mortality [1]. The onset of obesity, and thus in turn T2D, continues to occur at a progressively younger age. Obesity and T2D frequently coexist, with a strong bidirectional relationship and this coexistence suggests potential synergy for many adiposity-related complications, including cancer.

Up to 14% of all cancers are attributable to excess adiposity, with obesity now surpassing smoking as the most prevalent risk factor, driving rising cancer incidence globally [2–4]. At least 13 types of adiposity-related cancer have been reported (endometrial, colorectal, breast, oesophageal, renal, pancreatic, liver, myeloma, ovarian and thyroid cancers), although unpublished data suggests additional site-specific cancers are also adiposity-related, particularly in females [5]. Meta-analysis demonstrates an incremental association between weight gain and risk of adiposity-related cancers [6], and importantly, childhood obesity specifically appears to increase the risk of colorectal, endometrial and pancreatic cancers [7]. The obesity-associated risk is influenced greater by waist circumference than BMI, hence adiposity-related cancer [8]. Similarly, T2D is associated with an increased risk at similar cancer sites [9, 10], with cancer now the leading cause of mortality in patients with T2D, even exceeding that associated with cardiovascular disease (CVD) [11]. Given the rapidly rising incidence of early-onset obesity and T2D (defined by either pathology in a patient < 40 years), this population burden of adiposity-related cancer data is particularly concerning [12].

Patients commonly live with both obesity and T2D; however, the impact of their co-existence on adiposity-related cancer risk, compared to either risk factor alone, is not clear. Even considering obesity, different metabolic health subtypes mediate differential risks. Finally, to what extent this impacts risk of cancer in younger adults living with obesity and/or T2D is also unclear; it is biologically plausible that multiple mechanistic pathways are implicated, exaggerating the risk of adiposity-related cancer.

Therefore, the aims of this study were twofold: to assess (i) the additive/synergistic impact of living with obesity and T2D on adiposity-related cancer incidence, and (ii) if this risk extends to younger adults (whose cancer risk might be assumed to be smaller) with early-onset obesity and/or T2D.

## Methods

### Study design

We conducted a cohort study with anonymised data from TriNetX (TriNetX LLC, Cambridge, MA, USA), a global federated health research network with access to both inpatient and outpatient electronic medical records from health care organisations internationally, primarily secondary and tertiary care providers in North America and Western Europe. This analysis was conducted on the Global Collaborative Network, containing data from > 135 million patients with access to diagnoses, procedures, medications, laboratory values and genomic information worldwide. The data used in this study was collected on 7th June 2024. Further details on the network have been described elsewhere [13].

### Definitions

*Obesity* was defined using anthropometric assessments (body mass index (BMI) ≥ 30 kg/m^2^ or ICD-10 coding).

*T2D* was defined using biochemistry (HbA1c > 6.5%), ICD-10 coding, or prescription of glucose-lowering therapy (except for sodium-glucose co-transporter 2 inhibitors, and glucagon-like peptide-1 receptor agonists, which are approved for additional clinical indications: treatment of heart failure/chronic kidney disease, and obesity, respectively).

*No obesity*: Importantly, patients without obesity must have been free of ICD-10 coding for obesity and additionally have a BMI between 18.5 and 30 kg/m^2^.

### Population

We identified all adults (> 18 years) in TriNetX. Three cohorts were created, each cohort with an exposure arm and reference arm. Within the exposure arm, three cohorts were created: (i) cohort 1: obesity only, without T2D; (ii) cohort 2: T2D only, without obesity; and (iii) cohort 3: obesity and T2D. The reference arm was patients without obesity or T2D.

### Exclusion criteria

**Fig. 1:**
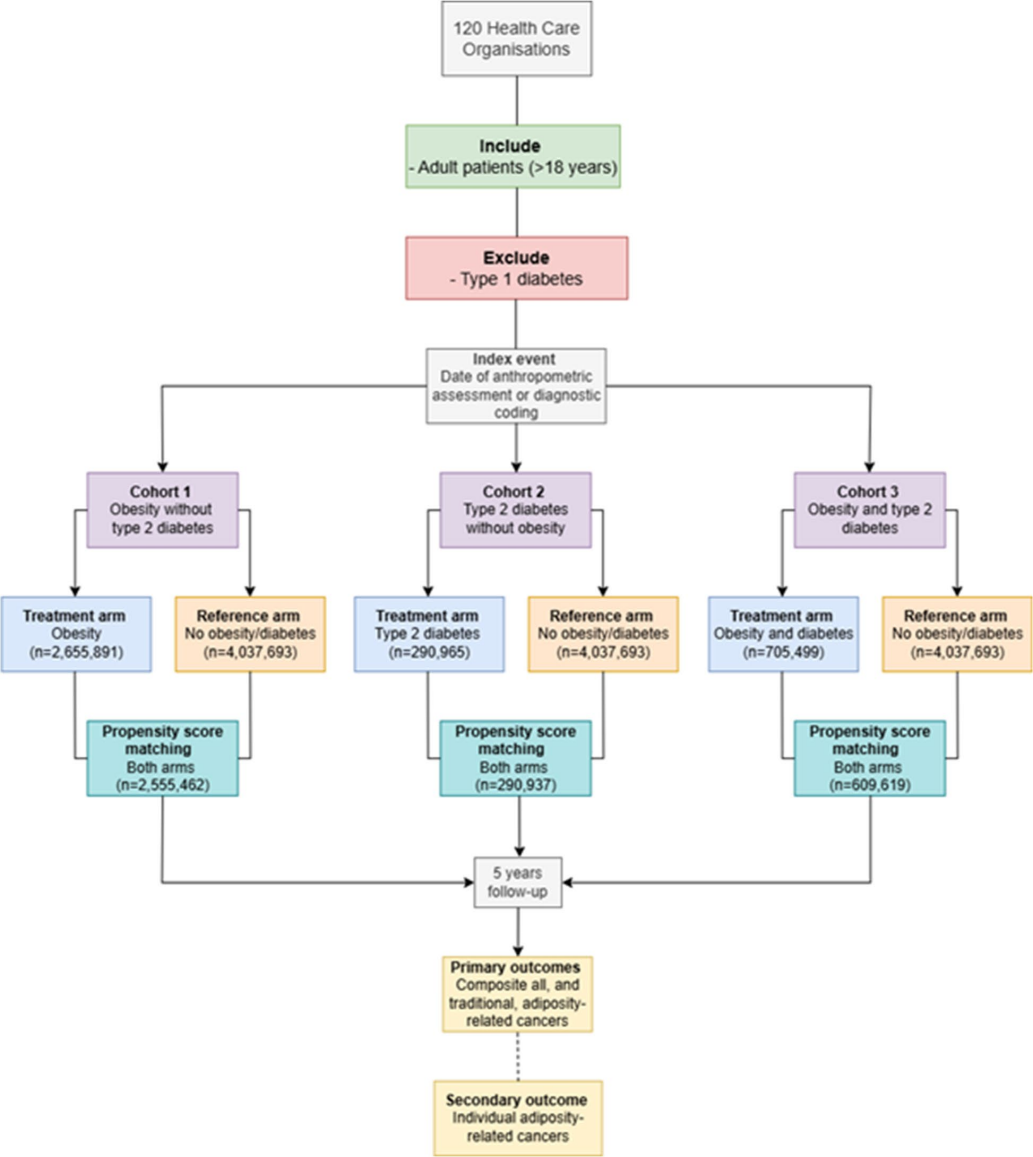
Consort diagram demonstrating inclusion and exclusion criteria during cohort creation

For all cohorts/arms, patients were excluded if they had a history of type 1 diabetes, or if, prior to the index event, they had been diagnosed with an adiposity-related cancer, as described by the European Society for Obesity (hepatocellular, colorectal, gastric, pancreatic, oesophageal, gallbladder, kidney, thyroid, brain, myeloma, breast, uterine, ovarian, oral cavity, nasal and sinus, adrenal, parathyroid, pituitary, connective tissue, head and neck, penis, melanoma, vulva, cervical) [14] (Fig. 1). Details for definitions of all diagnoses are presented in Additional File 1: Table S1.

### Index event

The index event for the exposure and reference arms followed either the date of diagnosis, via ICD-10 coding, or recording of BMI value. The index event was restricted to November 2018–2019. Patients were followed up for 5 years. The restricted dates for the index event were to ensure adequate propensity score matching (PSM) between arms could be achieved, as the TriNetX platform handles a capped volume of patient data dependent upon the number of covariates incorporated into the PSM model.

### Propensity score matching

Cohorts were PSM, in a 1:1 ratio, for age, sex, ethnicity, smoking, alcohol-related disorders, socioeconomic status, cardiovascular disease (CVD) (ischaemic heart disease (IHD), cerebrovascular accident (CVA) and peripheral vascular disease (PVD)), viral hepatitis, glomerular filtration rate (eGFR), presence of hypertension, dyslipidaemia, or previous non-obesity-related cancer. Additionally, cohort 2 had a PSM for BMI. Definitions for all PSM covariates are presented in Additional File 1: Table S1.

### Outcomes

*Primary outcome* was time-to-incident composite adiposity-related cancer. The composite outcome was grouped as all adiposity-related cancers (hepatocellular, colorectal, gastric, pancreatic, oesophageal, gallbladder, kidney, thyroid, brain, myeloma, breast, uterine, ovarian, oral cavity, nasal and sinus, adrenal, parathyroid, pituitary, connective tissue, head and neck, penis, melanoma, vulva, and cervical), in line with recent European Society for Obesity reports, and traditional adiposity-related cancers (hepatocellular, colorectal, gastric, pancreatic, oesophageal, gallbladder, kidney, thyroid, brain, myeloma, breast, uterine, and ovarian) [4]. *Secondary outcomes* of interest included time-to-incident of the individual adiposity-related cancers. All definitions used in the identification of outcomes are presented in Additional File 1: Table S1. Both arms were followed up until the first coding of the respective outcome of interest on their electronic medical records. Patients who did not develop incident cancer were censored at (i) the end of the time window for analysis, or (ii) the patient’s last known fact date; including any patient who had died during the analysis window.

### Statistical analysis

Statistical analysis for cohort data was performed in situ within the TriNetX platform. Normally distributed baseline characteristics are presented as mean and standard deviation (SD).

#### PSM

PSM was performed using logistic regression. TriNetX uses ‘greedy nearest-neighbour matching’ with a calliper of 0.1 pooled standard deviations and difference between propensity scores < 0.1. We assessed covariate balance between groups using the standardised mean difference (SMD). SMD < 0.1 was considered well-matched. The reference arm was considered the reference cohort (hazard ratio (HR) = 1) when compared against the exposure arm.

#### Survival analysis

Survival analysis was performed to estimate the probability of an outcome, at daily time intervals, over 12 months from the index event. Time-to-event analyses were conducted using Kaplan–Meier survival estimates and Cox proportional hazards models applied to the PSM cohorts, with exposure status included as the sole predictor. No additional covariates are included in the post-matching Cox model. A HR, log-rank test and Kaplan–Meier survival curve were generated. The proportional hazards assumption cannot be formally tested within the TriNetX platform (e.g. using Schoenfeld residuals); however, visual inspection of Kaplan–Meier curves can be undertaken. TriNetX uses the R Survival package v3.2–3. Additionally, for sensitivity analysis, we performed stratified analysis by age (> 60, < 60, and < 40 years), ethnicity (white *vs.* non-white ethnic background), and sex (male *vs.* female). We also performed a landmark analysis whereby we excluded incident cases of cancer within the first 12 months of the index event, and latterly directly compared the dual diagnosis of obesity and type 2 diabetes to obesity alone and type 2 diabetes alone.

#### *E*-values

Additionally, we calculated *E*-values, representing the minimum strength of association on the HR scale that an unmeasured confounder would need to have with both the exposure and the outcome, conditional on the measured confounders, to explain away the observed association; HR + √[HR × (HR − 1)] [15].

#### STROBE

The Strengthening the Reporting of Observational Studies in Epidemiology (STROBE) guidelines were followed in the reporting of this cohort study [16].

## Results

**Fig. 2:**
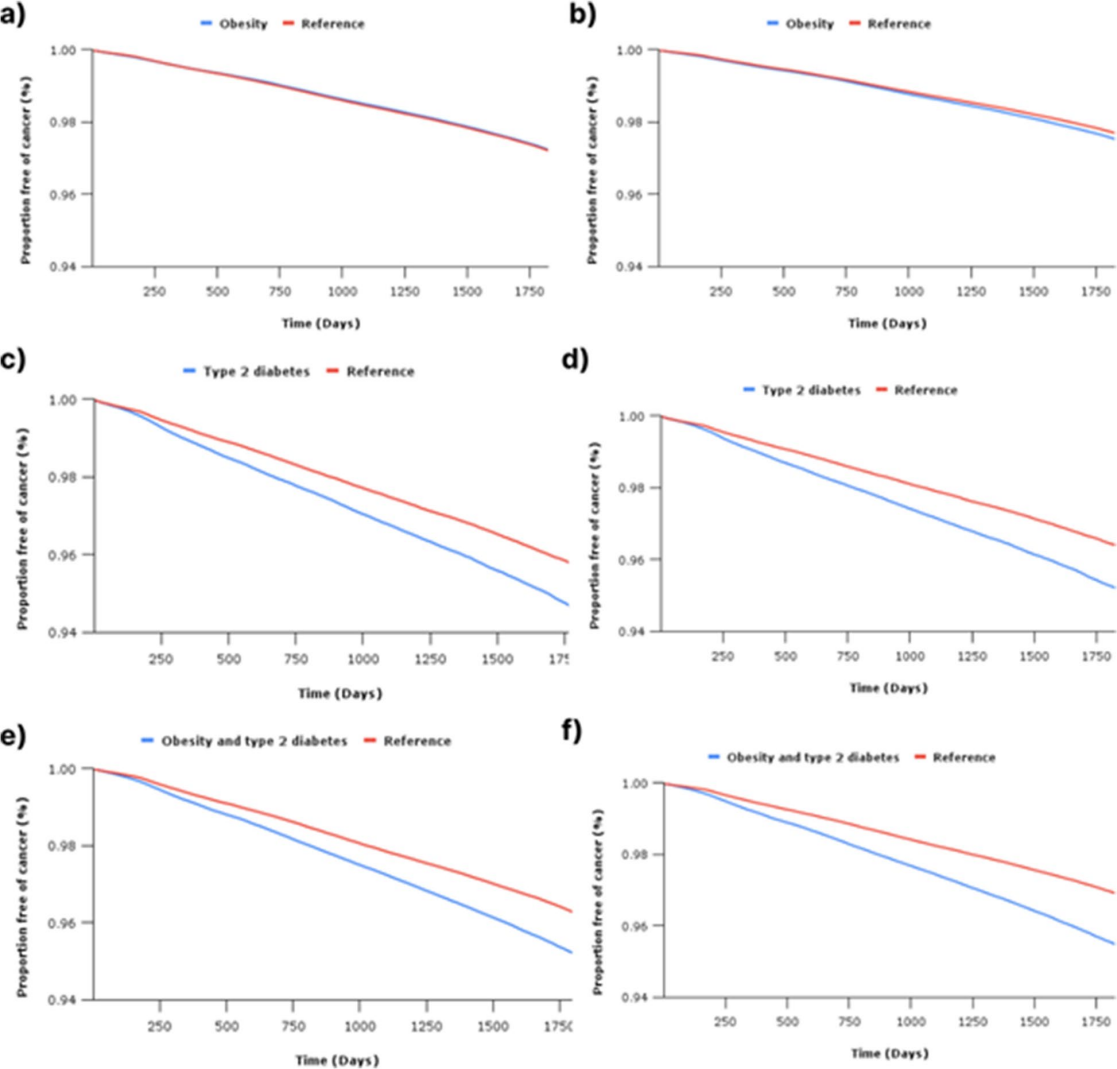
Kaplan Meier curves demonstrating results following survival analysis

**Fig. 3:**
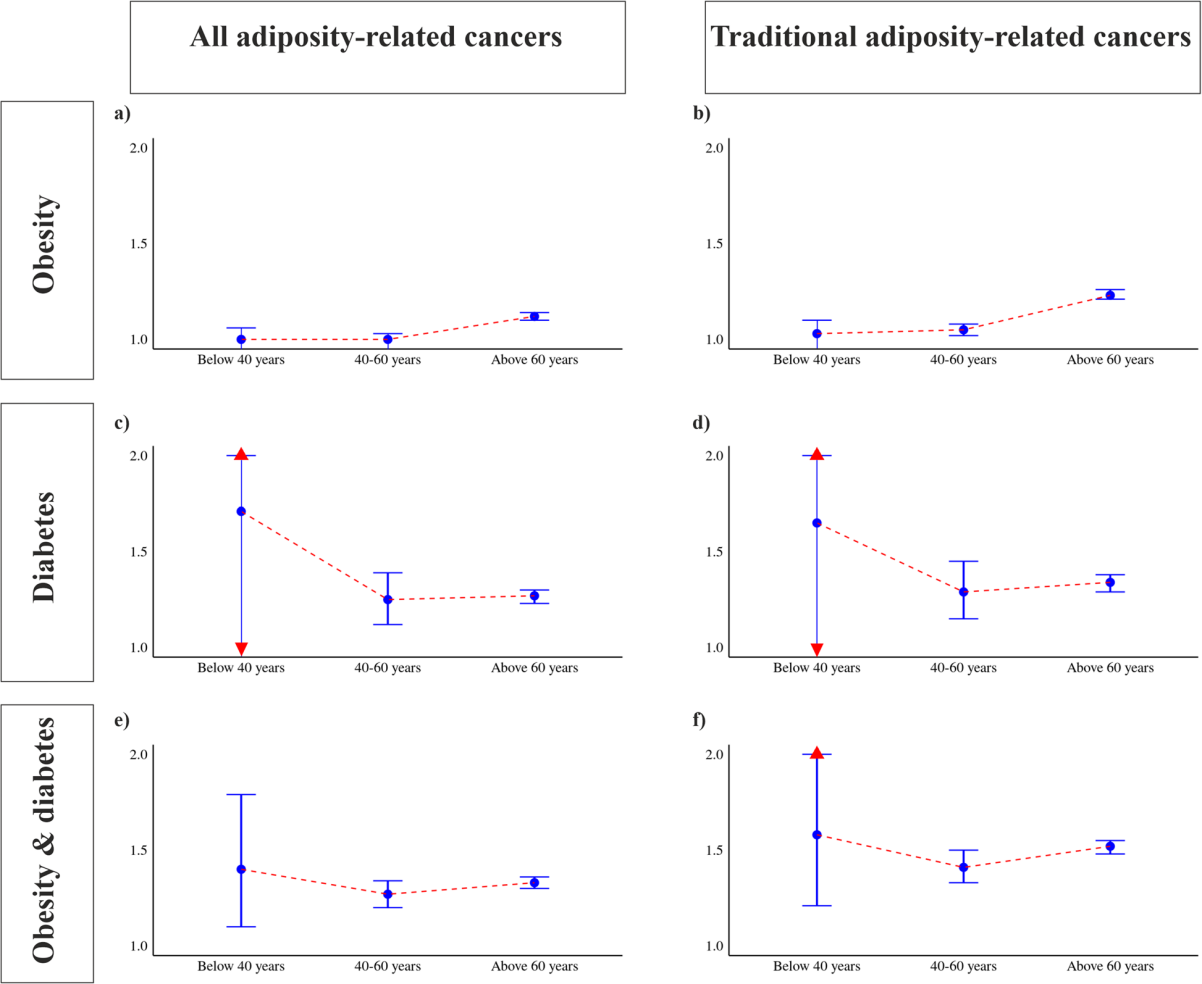
Forest plots reflecting the hazard ratios and 95% confidence intervals for stratified analyses by age

Baseline characteristics for all three cohorts are presented in Table 1, whilst Table 2, Additional File 1: Table S2–4, and Fig. 2 present the results of survival analysis for composite and individual adiposity-related cancer outcomes. The reference arm for all three cohorts included 4,037,693 patients without obesity or T2D. Stratified analyses are presented in Additional File 1: Table S5, with a detailed examination of the incidence of adiposity-related cancers by age presented in Fig. 3.

**Table 1:** Baseline characteristics for all three cohorts

	**Before propensity score matching**			**After propensity score matching**		
**Characteristic**	**Exposure**	**Reference**	**SMD**	**Treatment**	**Reference**	**SMD**
**Obesity cohort (cohort 1)**						
**Demographics**						
Numbers (n)	2,655,891	4,037,693		2,555,462	2,555,462	
Age (years)	48 ± 18	46 ± 22	0.08	48 ± 18	49 ± 19	0.09
Sex, female (%)	57.9	55.6	0.05	62.3	62.5	< 0.01
Ethnicity, white (%)	62.2	64.0	0.04	62.3	62.5	< 0.01
Adverse socioeconomic markers (%)	2.5	2.0	0.02	2.2	2.2	< 0.01
Smoking (%)	7.9	6.8	0.04	7.7	8.0	0.01
Alcohol-related disorders (%)	2.1	2.4	0.02	2.2	2.3	0.01
**Anthropometrics**						
Body mass index (kg/m^2^)	34.8 ± 6.0	24.1 ± 3.5	2.16	34.7 ± 6.0	24.5 ± 3.4	2.10
**Biochemistry**						
C-reactive protein (mg/L)	16.9 ± 37.8	15.5 ± 37.8	0.04	17.0 ± 37.9	15.2 ± 37.8	0.05
Glomerular filtration rate (ml/min/1.73 m^2^)	86.5 ± 26.7	86.3 ± 27.1	0.01	86.8 ± 26.8	86.2 ± 27.2	0.02
**Comorbidity (%)**						
Non-obesity-related cancer	16.2	16.2	< 0.01	16.3	16.2	< 0.01
Ischaemic heart disease	6.3	5.5	0.03	6.3	6.6	0.01
Cerebrovascular accident	3.3	3.7	0.02	3.4	3.7	0.01
Peripheral vascular disease	1.5	1.9	0.03	1.5	1.6	0.01
Viral hepatitis	0.9	1.1	0.02	1.0	1.0	0.01
Human immunodeficiency virus	0.4	0.5	0.02	0.4	0.6	0.03
Hypertension	27.2	16.8	0.25	24.4	24.6	0.01
Dyslipidaemia	23.2	17.4	0.14	21.8	22.3	0.01
**Medication (%)**						
Immunological agents	22.9	22.8	< 0.01	22.4	23.8	0.03
Anti-neoplastic agents	3.5	4.2	0.03	3.5	4.2	0.04
**Type 2 diabetes cohort (cohort 2)**						
**Demographics**						
Numbers (n)	290,965	4,037,693		290,937	290,937	
Age (years)	67.7 ± 13.1	46.2 ± 21.6	1.21	67.7 ± 13.1	68.2 ± 13.3	0.04
Sex, female (%)	42.3	55.5	0.27	42.3	41.7	0.01
Ethnicity, white (%)	51.1	63.7	0.26	51.1	51.5	0.01
Adverse socioeconomic markers (%)	2.2	2.1	< 0.01	2.2	2.0	0.01
Smoking (%)	10.6	7.2	0.12	10.6	10.6	< 0.01
Alcohol-related disorders (%)	3.1	2.5	0.03	3.1	3.1	< 0.01
**Anthropometrics**						
Body mass index (kg/m^2^)	25.9 ± 3.7	24.1 ± 3.5	0.52	25.9 ± 3.7	25.6 ± 3.3	0.09
**Biochemistry**						
HbA1c (%)	7.1 ± 1.7	5.7 ± 1.6	0.87	7.1 ± 1.7	5.9 ± 1.8	0.69
C-reactive protein (mg/L)	20.8 ± 45.6	15.2 ± 37.3	0.13	20.8 ± 45.6	17.8 ± 41.9	0.07
Glomerular filtration rate (ml/min/1.73 m^2^)	72.3 ± 29.2	85.4 ± 26.2	0.47	72.3 ± 29.2	74.4 ± 23.7	0.08
**Comorbidity (%)**						
Non-obesity-related cancer	24.6	16.9	0.19	24.6	24.4	0.01
Ischaemic heart disease	23.0	5.8	0.51	23.0	22.5	0.01
Cerebrovascular accident	13.0	3.9	0.33	13.0	12.8	0.01
Peripheral vascular disease	6.8	2.0	0.24	6.8	6.5	0.01
Viral hepatitis	2.7	1.2	0.11	2.7	2.4	0.02
Human immunodeficiency virus	0.6	0.6	0.01	0.6	0.8	0.02
Hypertension	61.4	17.2	1.01	61.4	60.7	0.01
Dyslipidaemia	56.6	17.4	0.89	56.6	55.8	0.02
**Medication (%)**						
Immunological agents	30.3	23.5	0.15	30.3	28.1	0.05
Anti-neoplastic agents	4.9	4.3	0.03	4.9	4.7	0.01
**Dual obesity and type 2 diabetes cohort (cohort 3)**						
**Demographics**						
Numbers (n)	705,499	4,037,693		609,619	609,619	
Age (years)	60.4 ± 13.4	46.4 ± 21.6	0.78	60.4 ± 14.0	61.5 ± 14.3	0.08
Sex, female (%)	50.6	55.6	0.10	50.7	48.7	0.04
Ethnicity, white (%)	60.7	64.0	0.07	62.8	62.2	0.01
Adverse socioeconomic markers (%)	3.0	2.0	0.06	2.6	2.5	0.01
Smoking (%)	13.2	6.8	0.21	12.4	12.5	< 0.01
Alcohol-related disorders (%)	3.0	2.4	0.04	3.3	3.2	< 0.01
**Anthropometrics**						
Body mass index (kg/m^2^)	36.4 ± 7.1	24.1 ± 3.5	2.20	36.3 ± 7.0	25.1 ± 3.3	2.06
**Biochemistry**						
HbA1c (%)	7.3 ± 1.8	5.7 ± 1.6	0.96	7.3 ± 1.8	5.7 ± 1.5	0.97
C-reactive protein (mg/L)	25.2 ± 49.2	15.5 ± 37.8	0.22	24.3 ± 47.9	26.4 ± 40.1	0.02
Glomerular filtration rate (ml/min/1.73 m^2^)	76.1 ± 28.7	86.3 ± 27.1	0.37	78.0 ± 27.6	77.6 ± 26.2	0.02
**Comorbidity (%)**						
Non-obesity-related cancer	26.3	16.1	0.25	26.4	24.8	0.04
Ischaemic heart disease	22.2	5.5	0.50	18.8	19.4	0.01
Cerebrovascular accident	9.8	3.7	0.24	9.4	9.3	< 0.01
Peripheral vascular disease	6.1	1.9	0.22	5.1	5.2	< 0.01
Viral hepatitis	1.9	1.1	0.07	1.9	1.8	< 0.01
Human immunodeficiency virus	0.5	0.5	< 0.01	0.5	0.9	0.05
Hypertension	69.8	16.6	1.27	65.0	64.9	< 0.01
Dyslipidaemia	61.6	17.4	1.01	55.8	54.8	0.02
**Medication**						
Immunological agents	34.1	22.8	0.25	32.8	30.2	0.06
Anti-neoplastic agents	4.0	4.2	0.01	3.9	5.2	0.06

**Table 2:** Results of survival analysis for all three cohorts

	**Sample size**	**Outcome (*n*)**	**5-year survival probability (%)**	**Hazard ratio (95% confidence interval)**	**Log-rank test**	***P* value**	***E*-value**
**Obesity**							
**All obesity-related cancers**							
Reference	2,555,462	45,582	97.2	1.00 (1.00–1.00)			
Obesity	2,555,462	45,370	97.2	0.99 (0.98, 1.01)	3.1	0.08	1.00
**Traditional obesity-related cancers**							
Reference	2,555,462	37,674	97.7	1.00 (1.00–1.00)			
Obesity	2,555,462	40,557	97.5	**1.07 (1.05, 1.09)**	89.0	< 0.01	1.34
**Type 2 diabetes**							
**All obesity-related cancers**							
Reference	290,937	8589	95.6	1.00 (1.00–1.00)			
Type 2 diabetes	290,937	11,207	94.5	**1.27 (1.23, 1.31)**	277.8	< 0.01	1.87
**Traditional obesity-related cancers**							
Reference	290,937	7055	96.4	1.00 (1.00–1.00)			
Type 2 diabetes	290,937	9775	95.2	**1.35 (1.31, 1.39)**	368.5	< 0.01	2.04
**Obesity and type 2 diabetes**							
**All obesity-related cancers**							
Reference	609,619	16,302	96.2	1.00 (1.00–1.00)			
Obesity and type 2 diabetes	609,619	21,766	95.1	**1.30 (1.27, 1.32)**	627.8	< 0.01	1.92
**Traditional obesity-related cancers**							
Reference	609,619	13,275	96.9	1.00 (1.00–1.00)			
Obesity and type 2 diabetes	609,619	20,187	95.5	**1.48 (1.45, 1.51)**	1237.2	< 0.01	2.32

### Cohort 1: Obesity without type 2 diabetes

There were 2,655,891 patients included in the obesity arm. Before matching, patients with obesity were more likely to have hypertension and dyslipidaemia. After PSM, the cohort was deemed well matched, and each arm included 2,555,462 patients.

#### Survival analysis

Obesity was not associated with an increased risk of all adiposity-related cancers (0.99 [0.98, 1.01]), although was associated with an increased risk of traditional adiposity-related cancers (1.07 [1.05, 1.09]). Specifically, obesity was associated with an increased risk of certain genitourinary and female reproductive system (vulval (1.18 [1.01, 1.37]), cervical (1.19 [1.09, 1.30]), uterine (2.21 [2.09, 2.34]), ovarian (1.13 [1.06, 1,21]), and kidney (1.31 [1.24, 1.37])), and endocrine glands (pituitary (1.49 [1.00, 2.22]), and thyroid (1.24 [1.18, 1.31])) cancers. However, obesity was associated with a lower risk of pancreatic (0.81 [0.76, 0.86]), oesophageal (0.86 [0.79, 0.94]), adrenal (0.70 [0.57, 0.87]), oral cavity (0.61 [0.54, 0.68]), head and neck (0.62 [0.58, 0.67]), brain (0.86 [0.80, 0.93]), and bone marrow (0.92 [0.87, 0.97) cancers, as well as melanoma (0.79 [0.75, 0.83]).

#### Stratified analyses

Stratified by age, the highest incidence of all (1.12 [1.10, 1.14]), and traditional (1.23 [1.21, 1.26]), adiposity-related cancers was found in older adults living with obesity. Patients of non-white ethnicity, living with obesity, had a reduced risk of all (0.91 [0.89, 0.93]), and traditional (0.97 [0.94, 0.99]), adiposity-related cancers, whilst patients of white ethnicity were at increased risk of traditional adiposity-related cancers (1.10 [1.08, 1.12]). Female sex carried a higher risk of incident all (1.04 [1.02, 1.06]), and traditional (1.12 [1.11, 1.14]), adiposity-related cancers. Finally, when the landmark analysis was performed, obesity alone was associated with a reduced risk of all adiposity-related cancers (0.94 [0.93, 0.95]), but an increased risk of traditional adiposity-related cancers (1.01 [1.00, 1.03]).

### Cohort 2: Type 2 diabetes without obesity

There were 290,965 patients in the T2D arm. Before matching, patients with T2D were more likely to be older, male, and of non-white ethnicity. They were more likely to smoke and be living with overweight, hypertension, dyslipidaemia, non-adiposity-related cancers, CVD, and viral hepatitis, as well as being prescribed more immunological therapy. Their biochemistry demonstrated worse renal function. After PSM, the cohort was deemed well-matched, and each arm included 290,937 patients.

#### Survival analysis

T2D was associated with an increased risk of all adiposity-related cancers (1.27 [1.23, 1.31]), and of traditional adiposity-related cancers (1.35 [1.31, 1.39]). Specifically, T2D was associated with an increased risk of certain gastrointestinal (hepatocellular (2.26 [2.04, 2.49]), colorectal (1.34 [1.25, 1.44]), pancreatic (2.07 [1.87, 2.29]), oesophageal (1.32 [1.14, 1.54]), and gastric (1.68 [1.45, 1.95]) cancers), genitourinary and female reproductive system (cervical (1.40 [1.09, 1.79]), uterine (1.27 [1.10, 1.48]), ovarian (1.28 [1.08, 1.52]), and kidney (1.37 [1.25, 1.50]) cancers), endocrine gland (thyroid (1.20 [1.04, 1.39]), adrenal (1.56 [1.06, 2.30]), and pituitary (3.63 [1.20, 10.92]) cancers). However, T2D was associated with a lower risk of melanoma (0.69 [0.61, 0.77]).

#### Stratified analyses

When stratified by age, younger adults had the highest, but insignificant, risk of all (1.71 [0.96, 3.06]), and traditional (1.65 [0.87, 3.14]), adiposity-related cancers. Patients of white ethnicity, living with T2D, had the highest risk of all (1.29 [1.24, 1.34]), and traditional (1.36 [1.30, 1.41]), adiposity-related cancers. Both male and female sexes had an increased risk of adiposity-related cancers. Finally, when the landmark analysis was performed, T2D alone was associated with an increased risk of both all adiposity-related cancers (1.25 [1.21, 1.28]), and traditional adiposity-related cancers (1.32 [1.28, 1.36]).

### Cohort 3: Obesity and type 2 diabetes

There were 705,499 patients included in the obesity and T2D arm. Before matching, patients with T2D were more likely to be older and male. They were more likely to smoke and be living with hypertension, dyslipidaemia, non-adiposity-related cancers, and CVD, as well as being prescribed more immunological therapy. Their biochemistry demonstrated more inflammation and worse renal function. After PSM, the cohort was deemed well matched, and each arm included 609,619 patients.

#### Survival analysis

Obesity and T2D were associated with an increased risk of all adiposity-related cancers (1.30 [1.27, 1.32]), and of traditional adiposity-related cancers (1.48 [1.145, 1.51]). Specifically, obesity and T2D were associated with an increased risk of certain gastrointestinal (hepatocellular (2.28 [2.11, 2.56]), colorectal (1.47 [1.39, 1.55]), gallbladder (1.62 [1.23, 2.13]), pancreatic (1.57 [1.45, 1.70]), and gastric (1.38 [1.22, 1.57]) cancers), genitourinary and female reproductive system (vulval (1.59 [1.27, 2.01]), cervical (1.67 [1.43, 1.95]), uterine (3.58 [3.27, 3.92]), breast (1.17 [1.12, 1.22]), ovarian (1.45 [1.29, 1.62]), penile (2.33 [1.60, 3.38]) and kidney (1.76 [1.65, 1.88]) cancers), endocrine gland (thyroid (1.45 [1.32, 1.59]), and pituitary (2.60 [1.37, 4.91]) cancers), connective tissue (1.22 [1.10, 1.35]), and bone marrow (1.20 [1.10, 1.31]) cancers. However, T2D was associated with a reduced risk of oral cavity (0.65 [0.55, 0.76]), and head and neck (0.66 [0.60, 0.74]) cancers, as well as melanoma (0.66 [0.60, 0.71]).

#### Stratified analyses

When stratified by age, younger adults had the highest risk of all (1.40 [1.10, 1.79]), and traditional (1.58 [1.21, 2.05]), adiposity-related cancers. Patients of white ethnicity, living with obesity, had the highest risk of all (1.34 [1.30, 1.37]), and traditional (1.54 [1.50, 1.58]), adiposity-related cancers. Both male and female sexes had an increased risk of adiposity-related cancers. Finally, when the landmark analysis was performed, dual diagnosis of obesity and T2D was associated with an increased risk of both all adiposity-related cancers (1.24 [1.22, 1.27]) and traditional adiposity-related cancers (1.40 [1.38, 1.43]).

When directly comparing a dual diagnosis of obesity and T2D to obesity alone, there was a significantly increased risk of all (1.22 [1.19, 1.24]), and traditional (1.27 [1.24, 1.30]), adiposity-related cancers in the dual diagnosis group. Moreover, when directly comparing a dual diagnosis of obesity and T2D to T2D alone, there was an increased risk of both all (1.02 [1.00, 1.05]), and traditional (1.08 [1.05, 1.12]), adiposity-related cancers.

## Discussion

Using a large, real-world database, we demonstrate a 30–48% higher incidence of adiposity-related cancer in people living with both obesity and type 2 diabetes. Specifically, this increased risk appears to be driven by site-specific gastrointestinal (hepatocellular, colorectal, gallbladder, pancreatic, and gastric), genitourinary and female reproductive system (vulval, cervical, uterine, breast, ovarian, penile, and kidney), endocrine gland (thyroid and pituitary), connective tissue, and bone marrow cancers. Over 5 years, the risk is greatest in female patients of white ethnicity, with early-onset type 2 diabetes complicating their obesity (under the age of 40). These results highlight the significant oncological risk of this patient cohort, particularly the younger cohort.

The pathophysiological abnormalities inherent in people living with obesity include excess adiposity, hyperinsulinaemia (with/without hyperglycaemia) and a pro-inflammatory environment, implicating obesity in progression of both type 2 diabetes and cancer. The International Agency for Research on Cancer (IARC) reports strong epidemiological evidence for an association between obesity and 13 cancers: breast, colorectal, endometrial, oesophageal, pancreatic, renal, liver, stomach, gallbladder, ovarian, thyroid, bone marrow, and meningioma [5]. One meta-analysis suggests the strongest associations are observed between obesity and endometrial and kidney cancer, whilst another demonstrates that for every 5 unit (kg/m^2)^ increase in BMI the risk of endometrial cancer increased by 59% [2, 17]. Moreover, meta-analysis demonstrates that childhood obesity specifically increases the risk of colorectal, endometrial, and pancreatic cancers [7]. These findings are in keeping with our analysis, whereby, in patients with obesity without T2D, endometrial, uterine, and kidney cancers were amongst the most incident cancers. The effect size for endometrial cancer in our study (HR 2.21) is comparable to the meta-analysis (HR 2.54), however, overall, we demonstrate weaker associations between obesity and most adiposity-related cancers. This diminished risk is likely explained by the fact that patients with obesity (cohort 1) were excluded if they ever had a diagnosis of T2D, meaning they are metabolically healthier than previous studies included in the meta-analysis [17]. This finding of patients with metabolically healthier obesity having a lower risk of cancer than their metabolically unhealthy counterparts has been shown previously [18]. Moreover, in ethnicity-stratified analyses, we observed attenuation of adiposity-related cancer risk among individuals of non-white ethnic background compared with white individuals. This finding is counterintuitive from a biological perspective, as several non-white populations, particularly South and East Asian groups, are known to develop greater visceral and ectopic adiposity, insulin resistance, and metabolic dysfunction at lower BMI thresholds [19], factors that would be expected to amplify rather than attenuate obesity-related cancer risk. The observed attenuation may therefore reflect methodological or epidemiological factors rather than true biological protection. Differences in cancer screening uptake, healthcare access, and diagnostic coding within real-world electronic health record datasets may influence recorded cancer incidence across ethnic groups. In addition, residual confounding by lifestyle factors and migration-related variables not fully captured in matching may contribute. Finally, competing risks and differential healthcare utilisation patterns may influence cancer detection within the study timeframe. These findings should therefore be interpreted cautiously and considered hypothesis-generating. However, ectopic fat deposition does still modulate cancer risk, highlighted by the association of MASLD with several extra-hepatic cancers [20].

Furthermore, meta-analysis demonstrates the association of T2D with similar sites of cancers to obesity. However, importantly, most obesity-associated site-specific cancers have a stronger association with T2D, as with our findings [17], and here, T2D drives carcinogenicity independent of BMI [21]. The largest meta-analysis of cohort studies (collecting data from 151 cohorts, comprising 32 million people) concluded a causal relationship between T2D and liver, pancreatic, and endometrial cancer incidence and pancreatic cancer mortality [22]; four site-specific cancers that were at increased risk following our analysis in patients with T2D without obesity. However, an umbrella review of meta-analyses of observational studies examined the association between T2D and cancer and debated some of these causal associations, suggesting that apart from certain site-specific gastrointestinal (cholangiocarcinoma and colorectal cancer) and female reproductive system (endometrial and breast cancer) cancers, previous research may be limited by residual bias confounding downstream of a diagnosis of T2D [23]. Despite this, Mendelian randomisation (MR) studies support the findings of observational work in highlighting genetic associations between hyperinsulinaemia and gastrointestinal (pancreatic) and female reproductive system and genitourinary (endometrial, breast, and renal cancer) cancers [24], together providing evidence for the robustness of our study results.

Finally, we suggest that obesity may have site-specific chemoprotection, notably with a lower risk of pancreatic, oesophageal, adrenal, oral cavity, head and neck, brain, and bone marrow cancers, and melanoma, supported by previous literature; although this has largely highlighted that obesity is protective against lung cancer which was not examined in the current study given our evaluation of proposed adiposity-related cancers [25].

The relationship between obesity, T2D and cancer is likely mediated via multiple distinct mechanisms. Five main theories support the association between obesity and cancer: (i) increased growth factor production, including insulin (hyperinsulinaemia) and insulin-like growth factor 1 (IGF-1); (ii) increased sex steroid hormones production, e.g. oestrogen; (iii) altered adipocytokines levels such as leptin, adiponectin, and visfatin, with growth, immune, and tumour regulatory properties; (iv) chronic low-grade inflammation and oxidative stress, upregulating growth-promoting cytokines and immune modulation; and (v) altered gastrointestinal microbiome diversity. Moreover, patients with T2D may have increased cancer risk through (i) hyperinsulinaemia induced stimulation of mitogenesis, production of IGF-1, and cellular metabolic activity resulting in DNA damage and mutagenesis; and (ii) hyperglycaemia induced stimulation of tumourigenesis (by providing the necessary metabolic fuel), and increased production of advanced glycation end products which drive oxidative stress and cell inflammation [26]. Finally, our finding that younger patients with T2D, but not with metabolically healthier obesity, are at increased risk of adiposity-related cancers may be expected. The Barker hypothesis suggests that adverse intrauterine and early-life environments can predispose individuals to metabolic dysfunction later in life. Younger patients diagnosed with early-onset T2D may therefore have experienced early metabolic programming and, combined with longer lifetime exposure to metabolic disturbances, may lack compensatory mechanisms to mitigate chronic metabolic dysfunction, resulting in greater susceptibility to cancer [27]. As a result of early-onset diabetes being associated with a more severe disease phenotype, it is plausible that such patients may have a higher associated healthcare utilisation (more frequent outpatient visits and more extensive biochemical testing and imaging) than the general population or those with uncomplicated obesity. This ‘detection bias’ may conceivably increase the likelihood of diagnosing earlier stage (asymptomatic) cancers that would otherwise have been undetected. To partially account for this, we matched for socioeconomic status and comorbidities, although we were unable to explicitly account for frequency of healthcare encounters prior to the index event within TriNetX. Other residual confounding, including diet quality and physical activity levels, should also be acknowledged as important confounders in the association of obesity and T2D with cancers. Future studies should aim to use data that have recorded such variables to account for these important sources of potential residual confounding.

A further limitation relates to the differential proportion of unmatched participants across analyses following PSM. In cohort 3, a substantially larger number of patients in the treatment arm were not retained after matching compared with cohorts 1 and 2. This reflects baseline differences in clinical characteristics between treatment groups in this analysis, resulting in a smaller region of common support and consequently a greater number of unmatched individuals. While PSM improves internal validity and strengthens causal inference by enhancing covariate balance between comparison groups, exclusion of unmatched participants may reduce external validity and introduce potential selection bias if retained participants differ systematically from those excluded. However, covariate balance after matching was deemed acceptable based on standardised mean differences, suggesting that the matched cohorts were well aligned for measured confounders. Findings from cohort 3 should therefore be interpreted in the context of this reduced matched sample size and potential for residual selection bias.

Moreover, the proportional hazards assumption could not be formally tested within the TriNetX platform (e.g. using Schoenfeld residuals). However, visual inspection of survival curves suggested no major violations.

We are currently witnessing dual pandemics of obesity and T2D. Given that both obesity (even with preserved metabolic health) and T2D (independent of obesity) appear to increase the risk of several site-specific cancers, it is unsurprising to observe the additive effect of the combined diagnoses. However, our age-stratified analysis, demonstrating amplified cancer risks in younger populations, provides stark insight to the population, public health experts and policymakers. Cancer incidence rates have increased most significantly in people < 50 years old; to illustrate, colorectal cancer, previously with a peak incidence of 67 years, is now increasingly presenting in people < 50 years according to the National Cancer Institute. Our results concur with findings from a recent longitudinal analysis which highlights that a younger age of T2D diagnosis was associated with higher incidence of overall and site-specific cancer. The Barker hypothesis provides the mechanistic basis for this phenomenon stating that younger patients are unable to develop compensatory mechanisms and deal with the burden of metabolic disturbance, thereby suffering worse outcomes [28]. Fortunately, with novel incretin-based therapies targeting glucagon-like peptide-1 (GLP-1), and glucose-dependent insulinotropic polypeptide (GIP), and producing weight loss of > 20% (approaching that seen following metabolic surgery), we are at a defining moment in our ability to treat the underlying obesity and T2D and their complications, and thus maybe mitigate this cancer risk. These medicines have already demonstrated potential benefit in reducing cancer incidence [29]. We, and others [30], would therefore suggest that early and lifelong treatment of obesity is critical for maintaining lean body mass as well as preventing weight recurrence, increased adiposity, and subsequent sequelae (in this case, adiposity-related cancers).

## Conclusions

The coexistence of obesity and type 2 diabetes is associated with the greatest increase in risk of incident adiposity-related cancers, supporting a synergistic interaction between these metabolic conditions in driving excess cancer risk. This excess risk was evident across all age groups, underscoring the importance of recognising combined metabolic dysfunction as a major oncogenic risk state. With the global rise in multimorbidity and earlier-onset of obesity and T2D, integrated strategies targeting both conditions are urgently needed to mitigate future cancer burden.

## Supplementary Information

Additional file 1.

## Data Availability

The data that support the findings of this study are available from TriNetX, LLC, https://trinetx.com/, but third-party restrictions apply to the availability of these data. The data were used under license for this study with restrictions that do not allow for the data to be redistributed or made publicly available. However, for accredited researchers, the TriNetX data are available for licensing at TriNetX, LLC. Data access may require a data sharing agreement and may incur data access fees. Data used in the generation of this paper was collected from the global TriNetX network and local data at LUHFT were not used.

## Abbreviations

BMI: Body mass index
CVA: Cerebrovascular accident
CVD: Cardiovascular disease
eGFR: Estimated glomerular filtration rate
GIP: Glucose-dependent insulinotropic polypeptide
GLP-1: Glucagon-like peptide-1
HbA1c: Glycated haemoglobin
HIPAA: Health Insurance Portability and Accountability Act
HR: Hazard ratio
IARC: International Agency for Research on Cancer
ICD-10: International Classification of Diseases, 10th Revision
IGF-1: Insulin-like growth factor 1
IHD: Ischaemic heart disease
LLC: Limited liability company
LUHFT: Liverpool University Hospitals NHS Foundation Trust
MA: Massachusetts
MASLD: Metabolic dysfunction-associated steatotic liver disease
MR: Mendelian randomisation
NHS: National Health Service
NICE: National Institute for Health and Care Excellence
PSM: Propensity score matching
PVD: Peripheral vascular disease
R: R statistical software
SD: Standard deviation
SMD: Standardised mean difference
STROBE: Strengthening the Reporting of Observational Studies in Epidemiology
T2D: Type 2 diabetes
UK: United Kingdom
USA: United States of America
