# Supplementary material for: Synergistic effects of obesity and type 2 diabetes on adiposity-related cancer risk across age strata
Source: BMC Med. 2026 Apr 13;24:312. doi: 10.1186/s12916-026-04842-8 (PMC13185421; doi:10.1186/s12916-026-04842-8)
Supplement: Supplementary file 1 — Additional file 1. [file 12916_2026_4842_MOESM1_ESM.docx]

**Additional file 1** Individual and synergistic effect of obesity and type 2 diabetes on the risk of adiposity-related cancers: impact of age, sex, and ethnicity in a large real-world data cohort.

**Contents**

- Page 2: **Additional File 1: Table S1** Definitions for all baseline diagnoses, covariates, and outcomes
- Pages 3-5: **Additional File 1: Table S2** Results of survival analysis for the individual adiposity-related cancer outcomes for patients with obesity vs those without obesity or type 2 diabetes
- Pages 6-8: **Additional File 1: Table S3** Results of survival analysis for the individual adiposity-related cancer outcomes for patients with type 2 diabetes vs those without obesity or type 2 diabetes
- Pages 9-11: **Additional File 1: Table S4** Results of survival analysis for the individual adiposity-related cancer outcomes for patients with obesity and type 2 diabetes vs those without obesity or type 2 diabetes
- Pages 12-13: **Additional File 1: Table S5** Stratified analyses for the outcomes of all, and traditional, adiposity-related cancers.

| **Diagnosis** | **ICD-10 code** |
| --- | --- |
| **Inclusion and exclusion criteria** | |
| Type 2 diabetes | E11 |
| Type 1 diabetes | E10 |
| Obesity | E66.0, E66.8, E66.9 |
| **Covariates** | |
| Hypertension | I10 |
| Dyslipidaemia | E78 |
| Nicotine dependence | F17.2 |
| Socioeconomic hazards | Z55-Z65 |
| Alcohol-use disorders | F10 |
| Ischaemic heart disease | I20-I25 |
| Cerebrovascular accidents | I60-I69 |
| Peripheral vascular disease | I73 |
| Viral hepatitis | B15-B19 |
| **Outcomes (cancer)** | |
| Oral cavity | C06 |
| Oesophageal | C15 |
| Gastric | C16 |
| Colorectal | C18-C19 |
| Hepatocellular | C22 |
| Gallbladder | C23 |
| Pancreas | C25 |
| Sinus | C31 |
| Connective tissue | C49 |
| Breast | C50 |
| Vulva | C51 |
| Cervix | C53 |
| Endometrium | C54 |
| Uterus | C55 |
| Ovary | C56 |
| Penis | C60 |
| Kidney | C64 |
| Brain | C71 |
| Thyroid | C73 |
| Adrenal | C74 |
| Parathyroid | C75.0 |
| Pituitary | C75.1 |
| Head and neck | C76 |
| Bone marrow | C90 |
| Melanoma | D03 |

**Additional File 1: Table S1** Definitions for all baseline diagnoses, covariates, and outcomes.

| **Additional File 1: Table S2** | **Sample size** | **Outcome (n)** | **5-year survival probability (%)** | **Hazard ratio (95% confidence interval)** | **Log-Rank test** | **P value** | **E-value** |
| --- | --- | --- | --- | --- | --- | --- | --- |
| **Obesity** | | | | | | | |
| **Gastrointestinal carcinomas** | | | | | | | |
| **Hepatocellular** | | | | | | | |
| Reference | 2,555,462 | 2265 | 99.9 | 1.00 (1.00, 1.00) | | | |
| Obesity | 2,555,462 | 2319 | 99.9 | 1.02 (0.96, 1.08) | 0.3 | 0.58 | 1.00 |
| **Colorectal** | | | | | | | |
| Reference | 2,555,462 | 5176 | 99.7 | 1.00 (1.00, 1.00) | | | |
| Obesity | 2,555,462 | 5341 | 99.7 | 1.03 (0.99, 1.07) | 1.6 | 0.21 | 1.00 |
| **Gallbladder** | | | | | | | |
| Reference | 2,555,462 | 184 | >99.9 | 1.00 (1.00, 1.00) | | | |
| Obesity | 2,555,462 | 223 | >99.9 | 1.20 (0.99, 1.46) | 3.5 | 0.06 | 1.00 |
| **Pancreatic** | | | | | | | |
| Reference | 2,555,462 | 2240 | 99.9 | 1.00 (1.00, 1.00) | | | |
| Obesity | 2,555,462 | 1820 | 99.9 | **0.81 (0.76, 0.86)** | 44.5 | <0.01 | 1.77 |
| **Oesophageal** | | | | | | | |
| Reference | 2,555,462 | 1135 | 99.9 | 1.00 (1.00, 1.00) | | | |
| Obesity | 2,555,462 | 984 | 99.9 | **0.86 (0.79, 0.94)** | 11.8 | <0.01 | 1.60 |
| **Gastric** | | | | | | | |
| Reference | 2,555,462 | 996 | 99.9 | 1.00 (1.00, 1.00) | | | |
| Obesity | 2,555,462 | 926 | 99.9 | 0.92 (0.84, 1.01) | 3.1 | 0.08 | 1.00 |
| **Genitourinary and female reproductive health carcinomas** | | | | | | | |
| **Vulval** | | | | | | | |
| Reference | 2,555,462 | 316 | >99.9 | 1.00 (1.00, 1.00) | | | |
| Obesity | 2,555,462 | 374 | >99.9 | **1.18 (1.01, 1.37)** | 4.5 | 0.03 | 1.64 |
| **Cervical** | | | | | | | |
| Reference | 2,555,462 | 909 | 99.9 | 1.00 (1.00, 1.00) | | | |
| Obesity | 2,555,462 | 1092 | 99.9 | **1.19 (1.09, 1.30)** | 15.4 | <0.01 | 1.67 |
| **Uterine and endometrium** | | | | | | | |
| Reference | 2,555,462 | 1709 | 99.9 | 1.00 (1.00, 1.00) | | | |
| Obesity | 2,555,462 | 3797 | 99.8 | **2.21 (2.09, 2.34)** | 778.3 | <0.01 | 3.85 |
| **Breast** | | | | | | | |
| Reference | 2,555,462 | 14,720 | 99.1 | 1.00 (1.00, 1.00) | | | |
| Obesity | 2,555,462 | 14,747 | 99.1 | 0.99 (0.97, 1.02) | 0.2 | 0.65 | 1.00 |
| **Ovarian** | | | | | | | |
| Reference | 2,555,462 | 1540 | 99.9 | 1.00 (1.00, 1.00) | | | |
| Obesity | 2,555,462 | 1757 | 99.9 | **1.13 (1.06, 1.21)** | 12.8 | <0.01 | 1.51 |
| **Penile** | | | | | | | |
| Reference | 2,555,462 | 107 | >99.9 | 1.00 (1.00, 1.00) | | | |
| Obesity | 2,555,462 | 102 | >99.9 | 0.95 (0.72, 1.24) | 0.16 | 0.69 | 1.00 |
| **Renal** | | | | | | | |
| Reference | 2,555,462 | 2952 | 99.8 | 1.00 (1.00, 1.00) | | | |
| Obesity | 2,555,462 | 3879 | 99.8 | **1.31 (1.24, 1.37)** | 119.5 | <0.01 | 1.95 |
| **Endocrine gland carcinomas** | | | | | | | |
| **Thyroid** | | | | | | | |
| Reference | 2,555,462 | 2808 | 99.8 | 1.00 (1.00, 1.00) | | | |
| Obesity | 2,555,462 | 3514 | 99.8 | **1.24 (1.18, 1.31)** | 74.0 | <0.01 | 1.79 |
| **Parathyroid** | | | | | | | |
| Reference | 2,555,462 | 19 | >99.9 | 1.00 (1.00, 1.00) | | | |
| Obesity | 2,555,462 | 30 | >99.9 | 1.57 (0.88, 2.79) | 2.4 | 0.12 | 1.00 |
| **Adrenal** | | | | | | | |
| Reference | 2,555,462 | 195 | >99.9 | 1.00 (1.00, 1.00) | | | |
| Obesity | 2,555,462 | 138 | >99.9 | **0.70 (0.57, 0.87)** | 10.2 | <0.01 | 2.21 |
| **Pituitary** | | | | | | | |
| Reference | 2,555,462 | 40 | >99.9 | 1.00 (1.00, 1.00) | | | |
| Obesity | 2,555,462 | 60 | >99.9 | **1.49 (1.00, 2.22)** | 3.9 | <0.05 | 2.35 |
| **Other carcinomas** | | | | | | | |
| **Oral cavity** | | | | | | | |
| Reference | 2,555,462 | 797 | >99.9 | 1.00 (1.00, 1.00) | | | |
| Obesity | 2,555,462 | 486 | >99.9 | **0.61 (0.54, 0.68)** | 77.7 | <0.01 | 2.66 |
| **Head and neck** | | | | | | | |
| Reference | 2,555,462 | 2022 | 99.9 | 1.00 (1.00, 1.00) | | | |
| Obesity | 2,555,462 | 1267 | 99.9 | **0.62 (0.58, 0.67)** | 178.9 | <0.01 | 2.61 |
| **Sinus** | | | | | | | |
| Reference | 2,555,462 | 144 | >99.9 | 1.00 (1.00, 1.00) | | | |
| Obesity | 2,555,462 | 119 | >99.9 | 0.82 (0.64, 1.05) | 2.6 | 0.11 | 1.00 |
| **Connective tissue** | | | | | | | |
| Reference | 2,555,462 | 1895 | 99.9 | 1.00 (1.00, 1.00) | | | |
| Obesity | 2,555,462 | 1797 | 99.9 | 0.94 (0.88, 1.00) | 3.3 | 0.07 | 1.00 |
| **Brain** | | | | | | | |
| Reference | 2,555,462 | 1611 | 99.9 | 1.00 (1.00, 1.00) | | | |
| Obesity | 2,555,462 | 1399 | 99.9 | **0.86 (0.80, 0.93)** | 16.5 | <0.01 | 1.60 |
| **Bone marrow** | | | | | | | |
| Reference | 2,555,462 | 2370 | 99.9 | 1.00 (1.00, 1.00) | | | |
| Obesity | 2,555,462 | 2192 | 99.9 | **0.92 (0.87, 0.97)** | 8.3 | <0.01 | 1.39 |
| **Melanoma** | | | | | | | |
| Reference | 2,555,462 | 3773 | 99.8 | 1.00 (1.00, 1.00) | | | |
| Obesity | 2,555,462 | 2990 | 99.8 | **0.79 (0.75, 0.83)** | 96.2 | <0.01 | 1.85 |

**Additional File 1: Table S2** Results of survival analysis for the individual adiposity-related cancer outcomes for patients with obesity *vs.* those without obesity or type 2 diabetes

| **Additional File 1: Table S3** | **Sample size** | **Outcome (n)** | **5-year survival probability (%)** | **Hazard ratio (95% confidence interval)** | **Log-Rank test** | **P value** | **E-value** |
| --- | --- | --- | --- | --- | --- | --- | --- |
| **Type 2 diabetes** | | | | | | | |
| **Gastrointestinal carcinomas** | | | | | | | |
| **Hepatocellular** | | | | | | | |
| Reference | 290,937 | 568 | 99.7 | 1.00 (1.00, 1.00) | | | |
| Type 2 diabetes | 290,937 | 1320 | 99.3 | **2.26 (2.04, 2.49)** | 277.3 | <0.01 | 3.95 |
| **Colorectal** | | | | | | | |
| Reference | 290,937 | 1264 | 99.4 | 1.00 (1.00, 1.00) | | | |
| Type 2 diabetes | 290,937 | 1745 | 99.1 | **1.34 (1.25, 1.44)** | 63.1 | <0.01 | 2.02 |
| **Gallbladder** | | | | | | | |
| Reference | 290,937 | 58 | >99.9 | 1.00 (1.00, 1.00) | | | |
| Type 2 diabetes | 290,937 | 67 | >99.9 | 1.12 (0.79, 1.59) | 0.4 | 0.53 | 1.00 |
| **Pancreatic** | | | | | | | |
| Reference | 290,937 | 560 | 99.7 | 1.00 (1.00, 1.00) | | | |
| Type 2 diabetes | 290,937 | 1193 | 99.4 | **2.07 (1.87, 2.29)** | 209.8 | <0.01 | 3.56 |
| **Oesophageal** | | | | | | | |
| Reference | 290,937 | 292 | 99.8 | 1.00 (1.00, 1.00) | | | |
| Type 2 diabetes | 290,937 | 399 | 99.8 | **1.32 (1.14, 1.54)** | 13.4 | <0.01 | 1.97 |
| **Gastric** | | | | | | | |
| Reference | 290,937 | 272 | 99.9 | 1.00 (1.00, 1.00) | | | |
| Type 2 diabetes | 290,937 | 471 | 99.8 | **1.68 (1.45, 1.95)** | 47.3 | <0.01 | 2.75 |
| **Genitourinary and female reproductive health carcinomas** | | | | | | | |
| **Vulval** | | | | | | | |
| Reference | 290,937 | 61 | >99.9 | 1.00 (1.00, 1.00) | | | |
| Type 2 diabetes | 290,937 | 75 | >99.9 | 1.19 (0.85, 1.67) | 1.0 | 0.31 | 1.00 |
| **Cervical** | | | | | | | |
| Reference | 290,937 | 106 | 99.9 | 1.00 (1.00, 1.00) | | | |
| Type 2 diabetes | 290,937 | 153 | 99.9 | **1.40 (1.09, 1.79)** | 7.2 | 0.01 | 2.15 |
| **Uterine and endometrium** | | | | | | | |
| Reference | 290,937 | 303 | 99.9 | 1.00 (1.00, 1.00) | | | |
| Type 2 diabetes | 290,937 | 398 | 99.8 | **1.27 (1.10, 1.48)** | 10.1 | <0.01 | 1.86 |
| **Breast** | | | | | | | |
| Reference | 290,937 | 2045 | 99.9 | 1.00 (1.00, 1.00) | | | |
| Type 2 diabetes | 290,937 | 2224 | 99.9 | 1.05 (0.99, 1.12) | 3.0 | 0.08 | 1.00 |
| **Ovarian** | | | | | | | |
| Reference | 290,937 | 227 | 99.9 | 1.00 (1.00, 1.00) | | | |
| Type 2 diabetes | 290,937 | 300 | 99.9 | **1.28 (1.08, 1.52)** | 7.9 | <0.01 | 1.88 |
| **Penile** | | | | | | | |
| Reference | 290,937 | 27 | >99.9 | 1.00 (1.00, 1.00) | | | |
| Type 2 diabetes | 290,937 | 38 | >99.9 | 1.36 (0.83, 2.23) | 1.54 | 0.22 | 1.00 |
| **Renal** | | | | | | | |
| Reference | 290,937 | 768 | 99.6 | 1.00 (1.00, 1.00) | | | |
| Type 2 diabetes | 290,937 | 1083 | 99.5 | **1.37 (1.25, 1.50)** | 44.5 | <0.01 | 2.08 |
| **Endocrine gland carcinomas** | | | | | | | |
| **Thyroid** | | | | | | | |
| Reference | 290,937 | 323 | 99.8 | 1.00 (1.00, 1.00) | | | |
| Type 2 diabetes | 290,937 | 400 | 99.8 | **1.20 (1.04, 1.39)** | 6.0 | 0.01 | 1.69 |
| **Parathyroid** | | | | | | | |
| Reference | 290,937 | 10 | >99.9 | 1.00 (1.00, 1.00) | | | |
| Type 2 diabetes | 290,937 | 10 | >99.9 | 1.93 (0.66, 5.65) | 1.5 | 0.22 | 1.00 |
| **Adrenal** | | | | | | | |
| Reference | 290,937 | 41 | >99.9 | 1.00 (1.00, 1.00) | | | |
| Type 2 diabetes | 290,937 | 66 | >99.9 | **1.56 (1.06, 2.30)** | 5.1 | 0.02 | 2.50 |
| **Pituitary** | | | | | | | |
| Reference | 290,937 | 10 | >99.9 | 1.00 (1.00, 1.00) | | | |
| Type 2 diabetes | 290,937 | 15 | >99.9 | **3.63 (1.20, 10.92)** | 6.0 | 0.01 | 6.72 |
| **Other carcinomas** | | | | | | | |
| **Oral cavity** | | | | | | | |
| Reference | 290,937 | 162 | 99.9 | 1.00 (1.00, 1.00) | | | |
| Type 2 diabetes | 290,937 | 204 | 99.9 | 1.22 (0.99, 1.50) | 3.6 | 0.06 | 1.00 |
| **Head and neck** | | | | | | | |
| Reference | 290,937 | 446 | 99.8 | 1.00 (1.00, 1.00) | | | |
| Type 2 diabetes | 290,937 | 469 | 99.8 | 1.02 (0.90, 1.16) | 0.08 | 0.78 | 1.00 |
| **Sinus** | | | | | | | |
| Reference | 290,937 | 32 | <99.9 | 1.00 (1.00, 1.00) | | | |
| Type 2 diabetes | 290,937 | 41 | >99.9 | 1.24 (0.78, 1.97) | 0.8 | 0.36 | 1.00 |
| **Connective tissue** | | | | | | | |
| Reference | 290,937 | 388 | 99.8 | 1.00 (1.00, 1.00) | | | |
| Type 2 diabetes | 290,937 | 430 | 99.8 | 1.07 (0.94, 1.23) | 1.0 | 0.31 | 1.00 |
| **Brain** | | | | | | | |
| Reference | 290,937 | 270 | 99.9 | 1.00 (1.00, 1.00) | | | |
| Type 2 diabetes | 290,937 | 311 | 99.8 | 1.12 (0.95, 1.31) | 1.8 | 0.19 | 1.00 |
| **Bone marrow** | | | | | | | |
| Reference | 290,937 | 581 | 99.7 | 1.00 (1.00, 1.00) | | | |
| Type 2 diabetes | 290,937 | 663 | 99.7 | 1.11 (0.99, 1.24) | 3.1 | 0.08 | 1.00 |
| **Melanoma** | | | | | | | |
| Reference | 290,937 | 727 | 99.6 | 1.00 (1.00, 1.00) | | | |
| Type 2 diabetes | 290,937 | 515 | 99.7 | **0.69 (0.61, 0.77)** | 43.4 | <0.01 | 2.26 |

**Additional File 1: Table S3** Results of survival analysis for the individual adiposity-related cancer outcomes for patients with type 2 diabetes *vs.* those without obesity or type 2 diabetes

| **Additional File 1: Table S4** | **Sample size** | **Outcome (n)** | **5-year survival probability (%)** | **Hazard ratio (95% confidence interval)** | **Log-Rank test** | **P value** | **E-value** |
| --- | --- | --- | --- | --- | --- | --- | --- |
| **Obesity and type 2 diabetes** | | | | | | | |
| **Gastrointestinal carcinomas** | | | | | | | |
| **Hepatocellular** | | | | | | | |
| Reference | 609,619 | 937 | 99.8 |  | | | |
| Obesity and type 2 diabetes | 609,619 | 2208 | 99.5 | **2.28 (2.11, 2.56)** | 471.1 | <0.01 | 3.99 |
| **Colorectal** | | | | | | | |
| Reference | 609,619 | 1995 | 99.5 | 1.00 (1.00, 1.00) | | | |
| Obesity and type 2 diabetes | 609,619 | 3026 | 99.3 | **1.47 (1.39, 1.55)** | 178.7 | <0.01 | 2.30 |
| **Gallbladder** | | | | | | | |
| Reference | 609,619 | 81 | >99.9 | 1.00 (1.00, 1.00) | | | |
| Obesity and type 2 diabetes | 609,619 | 136 | >99.9 | **1.62 (1.23, 2.13)** | 12.0 | <0.01 | 2.62 |
| **Pancreatic** | | | | | | | |
| Reference | 609,619 | 916 | 99.8 | 1.00 (1.00, 1.00) | | | |
| Obesity and type 2 diabetes | 609,619 | 1488 | 99.7 | **1.57 (1.45, 1.70)** | 117.1 | <0.01 | 2.52 |
| **Oesophageal** | | | | | | | |
| Reference | 609,619 | 515 | 99.9 | 1.00 (1.00, 1.00) | | | |
| Obesity and type 2 diabetes | 609,619 | 543 | 99.9 | 1.02 (0.90, 1.15) | 0.1 | 0.77 | 1.00 |
| **Gastric** | | | | | | | |
| Reference | 609,619 | 395 | 99.9 | 1.00 (1.00, 1.00) | | | |
| Obesity and type 2 diabetes | 609,619 | 566 | 99.9 | **1.38 (1.22, 1.57)** | 24.7 | <0.01 | 2.10 |
| **Genitourinary and female reproductive health carcinomas** | | | | | | | |
| **Vulval** | | | | | | | |
| Reference | 609,619 | 114 | >99.9 | 1.00 (1.00, 1.00) | | | |
| Obesity and type 2 diabetes | 609,619 | 188 | >99.9 | **1.59 (1.27, 2.01)** | 15.6 | <0.01 | 2.56 |
| **Cervical** | | | | | | | |
| Reference | 609,619 | 250 | 99.9 | 1.00 (1.00, 1.00) | | | |
| Obesity and type 2 diabetes | 609,619 | 433 | 99.9 | **1.67 (1.43, 1.95)** | 42.9 | <0.01 | 2.73 |
| **Uterine and endometrium** | | | | | | | |
| Reference | 609,619 | 596 | 99.9 | 1.00 (1.00, 1.00) | | | |
| Obesity and type 2 diabetes | 609,619 | 2206 | 99.5 | **3.58 (3.27, 3.92)** | 873.0 | <0.01 | 6.62 |
| **Breast** | | | | | | | |
| Reference | 609,619 | 4575 | 99.9 | 1.00 (1.00, 1.00) | | | |
| Obesity and type 2 diabetes | 609,619 | 5533 | 98.8 | **1.17 (1.12, 1.22)** | 61.4 | <0.01 | 1.62 |
| **Ovarian** | | | | | | | |
| Reference | 609,619 | 483 | 99.9 | 1.00 (1.00, 1.00) | | | |
| Obesity and type 2 diabetes | 609,619 | 723 | 99.8 | **1.45 (1.29, 1.62)** | 39.9 | <0.01 | 2.26 |
| **Penile** | | | | | | | |
| Reference | 609,619 | 39 | >99.9 | 1.00 (1.00, 1.00) | | | |
| Obesity and type 2 diabetes | 609,619 | 94 | >99.9 | **2.33 (1.60, 3.38)** | 20.9 | <0.01 | 4.09 |
| **Renal** | | | | | | | |
| Reference | 609,619 | 1367 | 99.7 | 1.00 (1.00, 1.00) | | | |
| Obesity and type 2 diabetes | 609,619 | 2487 | 99.4 | **1.76 (1.65, 1.88)** | 289.3 | <0.01 | 2.92 |
| **Endocrine gland carcinomas** | | | | | | | |
| **Thyroid** | | | | | | | |
| Reference | 609,619 | 750 | 99.8 | 1.00 (1.00, 1.00) | | | |
| Obesity and type 2 diabetes | 609,619 | 1121 | 99.8 | **1.45 (1.32, 1.59)** | 61.7 | <0.01 | 2.26 |
| **Parathyroid** | | | | | | | |
| Reference | 609,619 | 10 | >99.9 | 1.00 (1.00, 1.00) | | | |
| Obesity and type 2 diabetes | 609,619 | 14 | >99.9 | 1.93 (0.78, 4.79) | 2.1 | 0.15 | 1.00 |
| **Adrenal** | | | | | | | |
| Reference | 609,619 | 83 | >99.9 | 1.00 (1.00, 1.00) | | | |
| Obesity and type 2 diabetes | 609,619 | 74 | >99.9 | 0.86 (0.63, 1.18) | 0.9 | 0.35 | 1.00 |
| **Pituitary** | | | | | | | |
| Reference | 609,619 | 13 | >99.9 | 1.00 (1.00, 1.00) | | | |
| Obesity and type 2 diabetes | 609,619 | 35 | >99.9 | **2.60 (1.37, 4.91)** | 9.3 | <0.01 | 4.64 |
| **Other carcinomas** | | | | | | | |
| **Oral cavity** | | | | | | | |
| Reference | 609,619 | 334 | 99.9 | 1.00 (1.00, 1.00) | | | |
| Obesity and type 2 diabetes | 609,619 | 223 | >99.9 | **0.65 (0.55, 0.76)** | 26.1 | <0.01 | 2.45 |
| **Head and neck** | | | | | | | |
| Reference | 609,619 | 858 | 99.8 | 1.00 (1.00, 1.00) | | | |
| Obesity and type 2 diabetes | 609,619 | 589 | 99.9 | **0.66 (0.60, 0.74)** | 59.9 | <0.01 | 2.40 |
| **Sinus** | | | | | | | |
| Reference | 609,619 | 50 | >99.9 | 1.00 (1.00, 1.00) | | | |
| Obesity and type 2 diabetes | 609,619 | 45 | >99.9 | 0.87 (0.58, 1.30) | 0.5 | 0.50 | 1.00 |
| **Connective tissue** | | | | | | | |
| Reference | 609,619 | 678 | 99.8 | 1.00 (1.00, 1.00) | | | |
| Obesity and type 2 diabetes | 609,619 | 856 | 99.8 | **1.22 (1.10, 1.35)** | 14.9 | <0.01 | 1.74 |
| **Brain** | | | | | | | |
| Reference | 609,619 | 516 | 99.9 | 1.00 (1.00, 1.00) | | | |
| Obesity and type 2 diabetes | 609,619 | 520 | 99.9 | 0.97 (0.86, 1.10) | 0.2 | 0.67 | 1.00 |
| **Bone marrow** | | | | | | | |
| Reference | 609,619 | 965 | 99.8 | 1.00 (1.00, 1.00) | | | |
| Obesity and type 2 diabetes | 609,619 | 1197 | 99.7 | **1.20 (1.10, 1.31)** | 17.6 | <0.01 | 1.69 |
| **Melanoma** | | | | | | | |
| Reference | 609,619 | 1465 | 99.7 | 1.00 (1.00, 1.00) | | | |
| Obesity and type 2 diabetes | 609,619 | 995 | 99.8 | **0.66 (0.60, 0.71)** | 107.8 | <0.01 | 2.40 |

**Additional File 1: Table S4** Results of survival analysis for the individual adiposity-related cancer outcomes for patients with obesity and type 2 diabetes *vs.* those without obesity or type 2 diabetes

| **Stratification** | **Hazard ratio [95% confidence interval]** | |
| --- | --- | --- |
|  | **All adiposity-related cancers** | **Traditional adiposity-related cancers** |
| **Obesity** | | |
| **Age** | | |
| Older adults, >60 years | **1.12 [1.10, 1.14]** | **1.23 [1.21, 1.26]** |
| Middle aged adults, 40-60 years | 1.00 [0.97, 1.03] | **1.05 [1.02, 1.08]** |
| Younger adults, <40 years | 1.00 [0.94, 1.06] | 1.03 [0.96, 1.10] |
| **Ethnicity** | | |
| White | 1.01 [0.99, 1.03] | **1.10 [1.08, 1.12]** |
| Non-white | **0.91 [0.89, 0.93]** | **0.97 [0.94, 0.99]** |
| **Sex** | | |
| Male | 0.99 [0.97, 1.02] | **1.06 [1.03, 1.09]** |
| Female | **1.04 [1.02, 1.06]** | **1.12 [1.11, 1.14]** |
| **Landmark analysis** | | |
| 12 months after index | **0.94 [0.93, 0.95]** | **1.01 [1.00, 1.03]** |
| **Type 2 diabetes** | | |
| **Age** | | |
| Older adults, >60 years | **1.27 [1.23, 1.30]** | **1.34 [1.29, 1.38]** |
| Middle aged adults, 40-60 years | **1.25 [1.12, 1.39]** | **1.29 [1.15, 1.45]** |
| Younger adults, <60 years | 1.71 [0.96, 3.06] | 1.65 [0.87, 3.14] |
| **Ethnicity** | | |
| White | **1.29 [1.24, 1.34]** | **1.36 [1.30, 1.41]** |
| Non-white | **1.14 [1.09, 1.18]** | **1.20 [1.15, 1.25]** |
| **Sex** | | |
| Male | **1.23 [1.18, 1.28]** | **1.34 [1.28, 1.40]** |
| Female | **1.22 [1.17, 1.27]** | **1.25 [1.20, 1.31]** |
| **Landmark analysis** | | |
| 12 months after index | **1.25 [1.21, 1.28]** | **1.32 [1.28, 1.36]** |
| **Obesity and type 2 diabetes** | | |
| **Age** | | |
| Older adults, >60 years | **1.33 [1.30, 1.36]** | **1.52 [1.48, 1.55]** |
| Middle aged adults, 40-60 years | **1.27 [1.20, 1.34]** | **1.41 [1.33, 1.50]** |
| Younger adults, <60 years | **1.40 [1.10, 1.79]** | **1.58 [1.21, 2.05]** |
| **Ethnicity** | | |
| White | **1.34 [1.30, 1.37]** | **1.54 [1.50, 1.58]** |
| Non-white | **1.18 [1.14, 1.22]** | **1.31 [1.26, 1.36]** |
| **Sex** | | |
| Male | **1.23 [1.18, 1.27]** | **1.40 [1.35, 1.46]** |
| Female | **1.30 [1.26, 1.34]** | **1.46 [1.41, 1.50]** |
| **Landmark analysis** | | |
| 12 months after index | **1.24 [1.22, 1.27]** | **1.40 [1.38, 1.43]** |

**Additional File 1: Table S5** Stratified analyses for the outcomes of all, and traditional, adiposity-related cancer.
